# Virtual Histology to Evaluate Mechanisms of Pulmonary Artery Lumen Enlargement in Response to Balloon Pulmonary Angioplasty in Chronic Thromboembolic Pulmonary Hypertension

**DOI:** 10.3390/jcm9061655

**Published:** 2020-06-01

**Authors:** Wojciech Magoń, Jakub Stępniewski, Marcin Waligóra, Kamil Jonas, Roman Przybylski, Martyna Sikorska, Piotr Podolec, Grzegorz Kopeć

**Affiliations:** 1 Department of Cardiac and Vascular Diseases, Jagiellonian University Medical College, John Paul II Hospital, Prądnicka 80, 31-202 Krakow, Poland; magon.wojtek@gmail.com (W.M.); jakub.stepniewski@googlemail.com (J.S.); marcin.waligoora@gmail.com (M.W.); kamil.jns@gmail.com (K.J.); martyna.duplicka@gmail.com (M.S.); p.podolec@szpitaljp2.krakow.pl (P.P.); 2 Department of Heart Transplantation and Mechanical Circulatory Support, Centre for Heart Diseases, Wroclaw Medical University, Borowska 213, 50-556 Wrocław, Poland; przybylski240@gmail.com

**Keywords:** intravascular ultrasound, virtual histology, mechanism of balloon pulmonary angioplasty

## Abstract

Chronic thromboembolic pulmonary hypertension (CTEPH) results from an obstruction of pulmonary arteries (PAs) by organized thrombi. The stenosed PAs are targeted during balloon pulmonary angioplasty (BPA). We aimed to evaluate the mechanism of BPA in inoperable patients with CTEPH. We analyzed stenosed PAs with intravascular grey-scale ultrasound (IVUS) to determine the cross-sectional area (CSA) of arterial lumen and of organized thrombi. The composition of organized thrombi was assessed using virtual histology. We distinguished two mechanisms of BPA: Type A with dominant vessel stretching, and type B with dominant thrombus compression. PAs were assessed before (*n* = 159) and after (*n* = 98) BPA in 20 consecutive patients. Organized thrombi were composed of dark-green (57.1 (48.0–64.0)%), light-green (34.0 (21.4–46.4)%), red (6.4 (2.9–11.7)%;) and white (0.2 (0.0–0.9)%) components. The mechanism type depended on vessel diameter (OR = 1.09(1.01–1.17); *p* = 0.03). In type B mechanism, decrease in the amount of light-green component positively correlated with an increase in lumen area after BPA (*r* = 0.50; *p* = 0.001). The mechanism of BPA depends on the diameter of the vessel. Dilation of more proximal PAs depends mainly on stretching of the vessel wall while dilation of smaller PAs depends on compression of the organized thrombi. The composition of the organized thrombi contributes to the effect of BPA.

## 1. Introduction

Chronic thromboembolic pulmonary hypertension (CTEPH) is a rare and progressive disease caused by obstruction of pulmonary arteries (PAs) by organized thrombi with accompanying precapillary arteriopathy [1,2]. Unresolved thromboembolic material found in stenosed or occluded PA segments is not a uniform tissue and on histological examination forms a complex structure composed of a fresh thrombotic material and fibrotic tissue along with some amount of inflammatory components [3,4].

Currently pulmonary endarterectomy (PEA) remains the treatment of choice for most patients with CTEPH [5]. This procedure enables direct visualization of the organized thromboembolic material and provides tissue for additional microscopic and biochemical examination which contributes to prognostic stratification of operated patients [6]. Recently, a refined balloon pulmonary angioplasty (BPA) has been proposed as a treatment option for CTEPH patients who are not eligible for PEA [7,8,9]. The procedure requires balloon inflations in a diseased PA segments performed in a series of BPA sessions. As organized thrombi are not removed during BPA, investigation of its mechanisms is limited by a lack of tissue material. It has been postulated that vessel stretching [10] and intimal dissection [11] may, at least partly, explain the effects of BPA. However, the role of compression of organized thrombi has not been fully explored. Additionally, it has not been investigated whether the composition of organized thrombi affects the results of BPA [12,13].

The grey-scale intravascular ultrasound (IVUS) and the IVUS virtual histology (IVUS-VH) allow in-vivo assessment of the vessel wall. In coronary arteries, the dark-green and light-green colors represent fibrous and fibrofatty material, respectively. The red component corresponds to the necrotic core, whereas the white component is designated as calcium deposits. Based on our previous single case study we speculated that different components of organized thrombi present heterogeneous response to balloon inflation [14].

In the present study, in a group of inoperable CTEPH patients, we assessed whether compression of organized thrombi contributes to the mechanism of BPA. Additionally, we analyzed whether heterogeneity in the composition of organized thrombi may affect the result of BPA.

## 2. Materials and Methods

### 2.1. Study Population

For the present study, we recruited adult patients with inoperable CTEPH who were qualified for BPA treatment in our center, between 1 January 2015 and 30 December 2017. If patients were pretreated with PH targeted therapies, we required that they had been on a stable dose of these medications for at least three months before recruitment. The Jagiellonian University Ethics Committee approved the study protocol No 122.6120.237.2015, and written informed consent was obtained from each patient of the study group. All clinical investigations were conducted in line with the principles of the Declaration of Helsinki.

### 2.2. Evaluation of Patients

Diagnosis of CTEPH was established according to ESC guidelines, and the operability and eligibility for BPA were assessed for each patient by the local CTEPH team, consisting of a cardiac surgeon, an interventional cardiologist experienced in BPA, and a PH specialist [5,15,16]. In all patients, before starting BPA, we evaluated their World Health Organization functional class (WHO-FC), serum levels of the N-terminal prohormone of brain natriuretic peptide (NT-proBNP), and their 6-min walking distance (6MWD). Right heart catheterization was performed before and after every BPA session to obtain the mean pulmonary artery pressure (mPAP), right atrial pressure (RAP), pulmonary vascular resistance (PVR), and cardiac index (CI) [17,18].

### 2.3. Balloon Pulmonary Angioplasty

Every BPA session consisted of a series of balloon inflations (BPAs) at stenosed PAs segments as described in detail in our recent report [19].

### 2.4. Identification of Stenoses of PA Segments

The PA segments subjected to BPA were initially visualized with selective pulmonary angiography and the stenotic lesions were categorized, according to a recently devised method of classification, as either web lesions, ring lesions, subtotal occlusions, total occlusions, or tortuous lesions [12]. The presence of organized thrombi was confirmed using IVUS grey-scale imaging (Eagle Eye, Volcano, San Diego, CA, USA).

For further evaluation, we chose only the web and ring lesions, and excluded subtotal occlusions, total occlusions and tortuous lesions as we aimed to assess the lesions with IVUS before and directly after BPA. For the final analysis, we included only the lesions for each we had attained stabilization of the IVUS probe (free of forward and backward movements) both before and after BPA. To ascertain that the same lesion was assessed before and after BPA, we used anatomical landmarks and IVUS imaging.

### 2.5. IVUS Evaluation of PA Stenoses 

**Figure 1:**
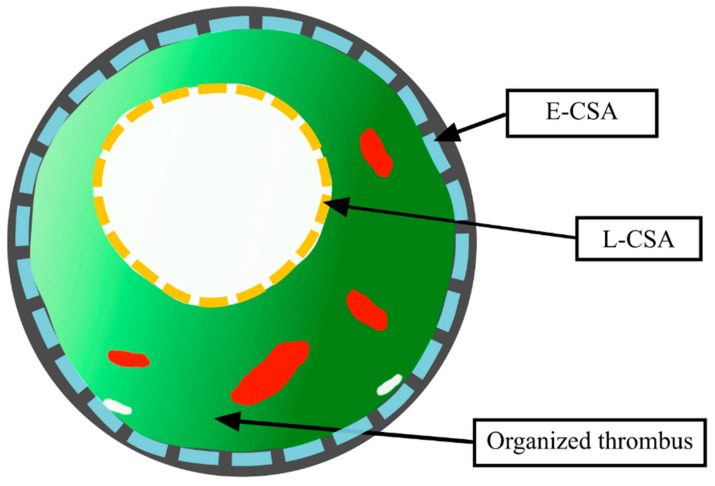
Schematic presentation of the pulmonary artery (PA) segment occupied by an organized thromboembolic lesion The external elastic membrane is depicted with the grey color. Dashed blue line enclosed by the external elastic membrane constituted the overall segment cross-sectional area (E-CSA in the text). The lumen of the stenosed PA segment (L-CSA in the text) is depicted by yellow dashed line.

PA segments occupied by the organized thrombi (web and ring-like) were visualized in both IVUS grey-scale imaging and IVUS-VH imaging (Eagle Eye, Volcano, San Diego, CA, USA). In recorded grey-scale image loops, we manually circumscribed off-line the lumen of the stenosed PA segment (lumen cross-sectional area; L-CSA) and the area enclosed by the external elastic membrane (E-CSA), as schematically presented in Figure 1 and measured the vessel diameter.

The CSA of the organized thrombi (T-CSA) was calculated as the difference between the E-CSA and the L-CSA. Measurements were obtained during diastole as the average value from over three consecutive cardiac cycles. Dedicated software (Eagle Eye, Volcano, San Diego, CA, USA) was used to describe the heterogeneity in the structure of the organized thrombi. Virtual histology, based on spectral analysis of the backscattered signal, marks different components of the assessed structure with either a dark-green, light-green, red, or white color. We measured the areas of each component of the organized thrombi, and presented them as a percentage of the T-CSA. The areas of different components of 15 consecutive organized thrombi were measured twice by WM in order to assess intra-observer variability, and by WM and JS to assess inter-observer variability.

### 2.6. Mechanisms of PA Lumen Enlargement in Response to BPA

Based on currently available evidence, we assumed that enlargement of the PA lumen in response to BPA might be as a result of either stretching of the PA wall (a well-described mechanism in clinical studies [10]) or, alternatively, as a result of compression of the organized thrombi (a proposed mechanism based on case studies [14]). Accordingly, we classified the BPA mechanism into two types: Type A with dominant vessel stretching, and type B without dominant vessel stretching, based on the median increase of the E-CSA (Δ E-CSA). This classification was carried out as follows:

1. **BPA mechanism Type A (dominant vessel stretching)**—defined as the ∆ E-CSA ≥ the median value; here the presumed main mechanism of BPA was vessel stretching;
2. **BPA mechanism Type B (non-dominant vessel stretching)**—defined as the ∆ E-CSA < the median value; the presumed main mechanism of BPA was different from that of vessel stretching.

### 2.7. Statistics

The categorical variables are presented as counts and percentages, and the continuous variables as medians and interquartile ranges. To assess differences between web and ring-type lesions, and between type A and type B mechanisms of BPA, we used the Mann–Whitney U test for continuous variables, and the *χ*^2^ test for categorical variables. To assess differences in continuous variables between the same lesions before and after BPA, we used the Wilcoxon matched-pairs signed-rank test. To analyze the determinants of the BPA mechanism, we performed multivariable logistic regression analysis, with the type A/type B response (1—type A, 0—type B) as the dependent variable, and E-CSA, T-CSA, the amount of light-green component and the amount of dark-green component as independent variables. To analyze the association between an increase in the L-CSA, and changes in amount of different components of organized thrombus, we used the Spearman correlation test. Inter-observer and intra-observer variability in the measurements of organized thrombi structure were assessed using the method of Bland and Altman [20]. Our previous analysis [14] showed that the decrease of thrombus area after BPA in the type B group was approximately 5 ± 8 mm^2^. Therefore, we assumed that that the mean difference in a change of thrombus CSA between type A and type B group will be 5 ± 8 mm^2^. Based on this assumption we calculated that for the 80% statistical power, the number of measurements needed to show a difference in Δ T-CSA between type A and type B groups would be 41 in each group. Therefore we planned to include approximately 100 PA segments into the analysis. To adjust for multiple comparisons, we used Bonferroni correction. The significance level was set at *p* < 0.05. The statistical analysis was performed with Stata/SE 12.1 (StataCorp LLC, College Station, TX, USA) and RStudio version 0.99.467 (RStudio PBC, Boston, MA, USA).

## 3. Results

### 3.1. Study Group

We analyzed 159 stenosed PA segments in a group of 20 patients with CTEPH. Twelve (60%) patients had been treated with stable doses of targeted medications before starting the first BPA session, including subcutaneous treprostinil (*n* = 5; 25%), riociguat (*n* = 4; 20%), or sildenafil (*n* = 1; 5%), or a combination of subcutaneous treprostinil and riociguat (*n* = 2; 10%). Eight patients had not been pretreated with targeted therapies before BPA as these therapies were not available at that time. One (5%) patient had persistent CTEPH after PEA. The baseline characteristics of enrolled patients are presented in Table 1.

**Figure 2:**
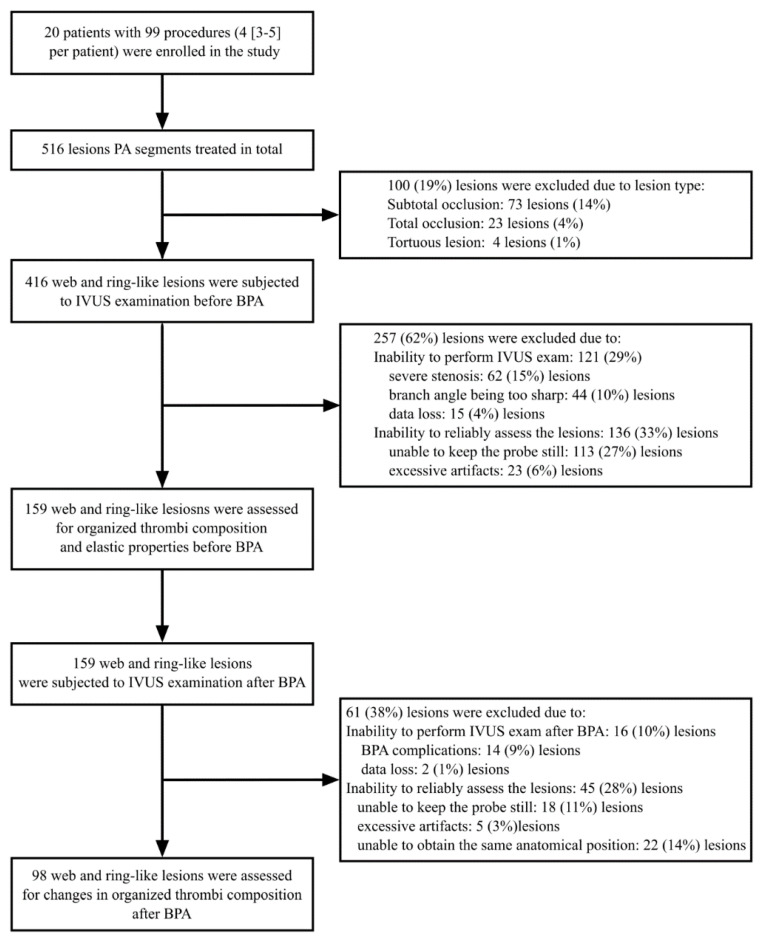
Flowchart presenting the selection of the stenosed pulmonary arterial segments. Abbreviations: BPA—balloon pulmonary angioplasty; IVUS—intravascular ultrasound.

Before performing BPA, we analyzed the structure of organized thrombi by IVUS-VH, finding that there were 25 (16%) ring-like lesions and 134 (84%) web lesions. The mechanism of BPA was assessed in 98 PA stenoses (ring-like stenosis: *n* = 13 (13%); web lesions: *n* = 85 (87%)) which we were able to visualize properly both before and immediately after BPA. In Figure 2, we show our protocol for PA segment selection.

### 3.2. Baseline Morphology of Stenosed PA Segments and Organized Thrombi

Of the 159 stenosed PA segments, 56 (35%) were located in segmental and 103 (65%) in subsegmental PA branches, both in the right lung (*n* = 102; 64%), and in the left lung (*n* = 57; 36%). 

The stenosed PA segments had a diameter of 4.7 (3.8–6.2) mm and an E-CSA of 13.5 (9.2–20.1) mm^2^. The T-CSA and L-CSA accounted for 72 (57–76)% and 28 (24–43)% of the E-CSA, respectively.

In IVUS-VH, we observed that the main component of the organized thrombi was dark-green, occupying 57.1 (48.0–64.0)% (3.6 (1.8–6.3) mm^2^) of the T-CSA. Next was the light-green component (34.0 (21.4–46.4)%; 2.2 (1.1–4.4) mm^2^), followed by the red component (6.4 (2.9–11.7)%; 0.4 (0.1–1.0) mm^2^), and finally, the white component (0.2 (0.0–0.9]%; 0.01 (0.00–0.06) mm^2^). The Bland–Altman chart (Figure S1) showed high intra-observer and inter-observer agreement between the measurements obtained for the four different components of the organized thrombi. The intraobserver coefficient of variance was 2.7 (2.6–2.9)%; 1.9 (1.8–1.9)%; 5.3 (4.9–5.6)%; 5.7 (5.1–6.4)% for dark-green, light-green, red and white component, respectively. The interobserver coefficient of variance was 5.8 (5.2–6.3)%; 2.6 (2.5–2.7)%; 5.9 (5.4–6.3)%; 13.9 (11.1–16.9)% for dark-green, light-green, red, and white component, respectively. There was no difference between ring and web-type lesions in terms of the composition of the organized thrombi (55.7 (49.3–63.0) vs. 56.0 (47.4–64.9)%; *p* = 0.88 for dark-green, 34.7 (26.8–41.1) vs. 35.1 (21.4–46.8)%; *p* = 0.99 for light green; 6.3 (3.6–10.7) vs. 6.4 (2.9–13.8)%; *p* = 0.91 for red and 0.2 (0.0–0.5) vs. 0.2 (0.0–0.9)%; *p* = 0.99 for the white component, respectively). Additionally we did not find any association between thrombus structure and pulmonary specific therapies (Table S1) or hemodynamic severity of CTEPH (Table S2). A schematic presentation of a PA segment occupied by an organized thromboembolic lesion is shown in Figure 1.

### 3.3. BPA Induced Changes of the Stenosed PA Segments

**Figure 3:**
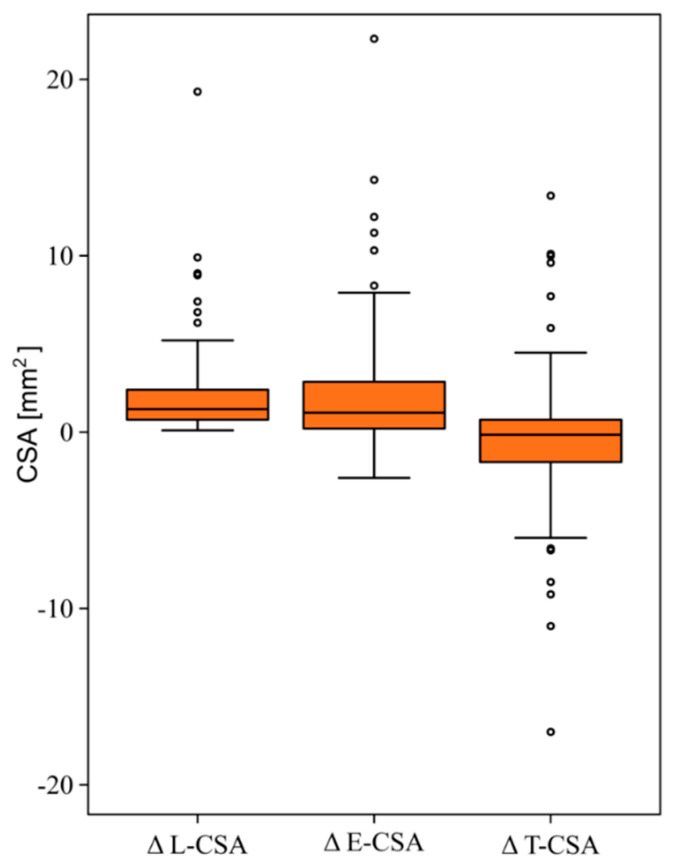
Distribution of changes in cross-sectional area (CSA) of the lumen, pulmonary artery stenosed segment and the organized thrombus after balloon pulmonary angioplasty. (*n* = 98). Both L-CSA and the E-CSA increased (∆ = 1.4(0.7–2.4) mm^2^; *p* < 0.001 and ∆ = 1.0(0.1–2.8) mm^2^; *p* < 0.001), respectively). There was no change in the T-CSA (∆ = −0.1(−1.3; 0.7) mm^2^; *p* = 0.23). The lines and boxes represent medians and interquartile (IQR) ranges. The width of the whisker is calculated as a 2*IQR. The points represent values outside the range denoted by whiskers. Abbreviations: E-CSA—pulmonary artery stenosed segment cross-sectional area; L-CSA—lumen cross-sectional area; T-CSA—organized thrombus cross-sectional area.

After balloon inflation, for all lesions, we observed an increase in the L-CSA (from 4.3 (2.9–5.7) to 5.9 (4.2–8.1) mm^2^; *p* < 0.001), and the E-CSA (from 13.6 (9.7–20.2] to 14.8 (10.8–24.3) mm^2^; *p* < 0.001). The median value of the T-CSA did not change after balloon inflations (9.2 (5.9–15.0) vs. 8.7 (5.5–13.9) mm^2^; *p* = 0.23). The effects were similar in ring and web-lesions (*p* = 0.74 for L-CSA; *p* = 0.57 for E-CSA, and *p* = 0.51 for T-CSA). However, we noted a wide distribution in relation to the T-CSA changes (∆ = −0.2 (−1.3; +0.6) mm^2^). In 45 (47%) stenosed PA segments, the T-CSA increased (from 0 to 9.6 mm^2^), and in 51 (53%) decreased (from 0 to −11.0 mm^2^) immediately after BPA. The distribution of the changes in the L-CSA, E-CSA, and T-CSA is shown in Figure 3. The median increase in the E-CSA (∆ E-CSA) after BPA was 1.0 (0.1–2.7) mm^2^. Based on this value, we distinguished the type A BPA mechanism: ∆ E-CSA ≥ 1.0 mm^2^ and type B mechanism: ∆ E-CSA < 1 mm^2^. One patient presented only Type A response in all assessed PA segments, while in the remaining patients we observed both types (A and B) of responses (60 (33–67)% of Type A response per patient).

### 3.4. Characterization of Type A and Type B Mechanisms of BPA

In the group with dominant stretching, type A, we observed an increase in E-CSA, which was in contrast to the non-dominant stretching group, type B (see Table 2). The L-CSA increased more in the type A group as compared to the type B group, while T-CSA decreased in the type B group and remained unchanged in the type A group. In Table 2, we compare the type A and type B groups with respect to the characteristics of the stenosed PA segments.

We found that the type A group, as compared to the type B group, was characterized by larger PA segments, larger organized thrombi, and had more light-green and less dark-green components. In the multivariable logistic regression analysis, the type A mechanism was predicted only by a larger E-CSA (Table 3).

### 3.5. Changes in the Structure of Organized Thrombi after BPA

**Figure 4:**
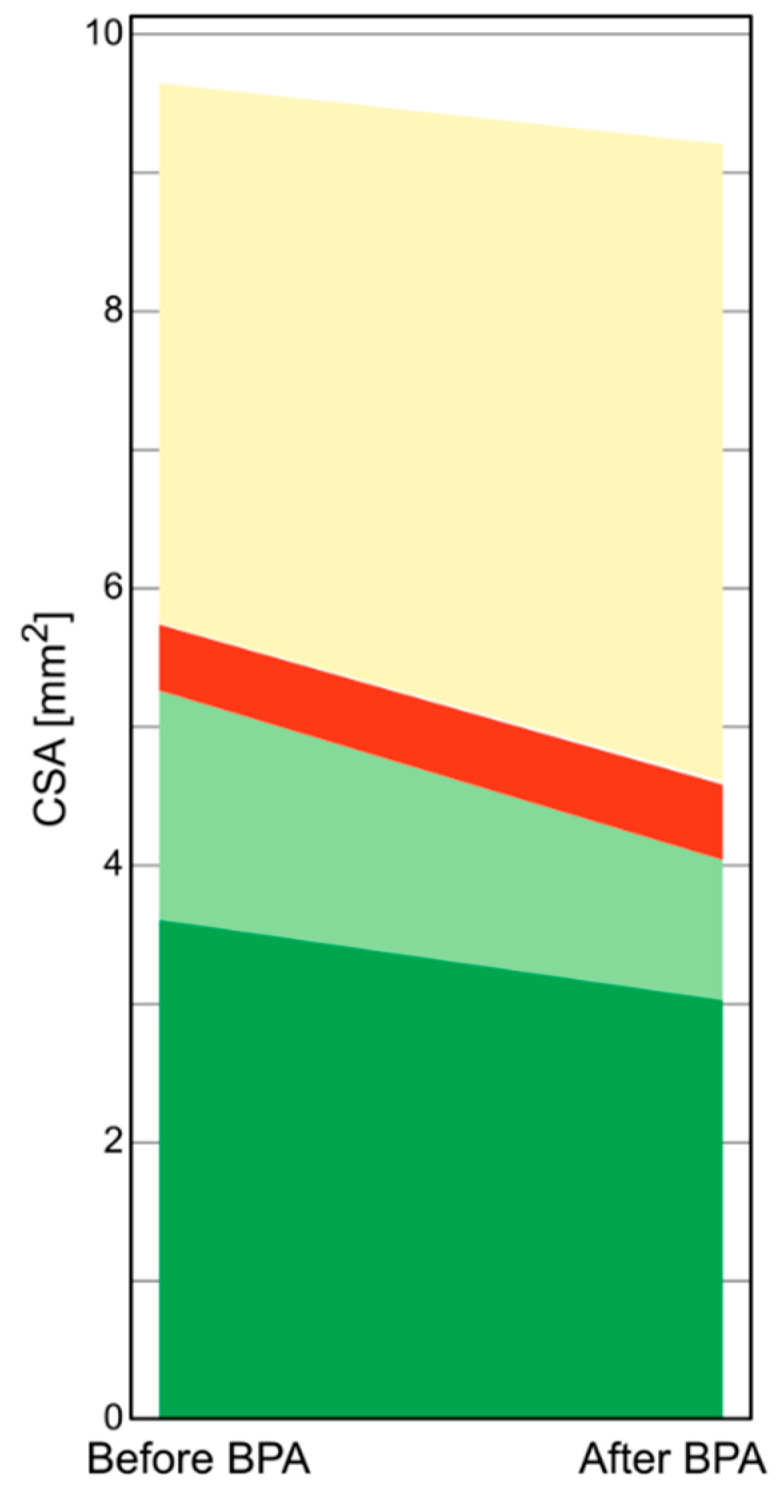
Changes in the organized thrombus composition in the type B (*n* = 49) response to BPA. There was an overall decrease in the T-CSA by 0.9 (−2.0; −0.1) mm^2^; *p* = 0.001. The L-CSA increased by 0.8 (0.6–1.7) mm^2^; *p* = 0.001 along with the decrease in the amount of light-green component (0.4 (−1; 0] mm^2^; *p* = 0.005). There was no significant change in the dark-green (∆ = −0.15 (−0.8; 0.3) mm^2^; *p* = 0.07), red (∆ = 0 (−0.2; 0.3) mm^2^; *p* = 0.65) and white (∆ = 0 (0; 0.1) mm^2^; *p* = 0.56) components of the organized thromboembolic lesions. Abbreviations: BPA—balloon pulmonary angioplasty; CSA—cross-sectional area; L-CSA—lumen cross-sectional area; T-CSA—organized thrombus cross-sectional area.

In Figure 4, we present a diagram which shows changes in organized thrombi structure in the type B mechanism. We observed a significant decrease in the CSA of the light-green component and no change in the CSA of other components. The decrease in the amount of light-green component positively correlated with the increase in the lumen area after BPA in the type B group (*r* = 0.50; *p* = 0.001).

## 4. Discussion

In the present study, in a group of inoperable CTEPH patients, we have expanded understanding of BPA mechanisms by identifying the second (type B) mechanism which contributes to enlargement of the vessel lumen in response to BPA. The first mechanism (type A) works by stretching the PA wall and is typical for larger PA segments, whereas the second mechanism (type B) is characterized by compression of the organized thrombus, and is more typical for PA segments of smaller diameters. Additionally, we have shown that the composition of a thrombotic lesion influences the effects of BPA; in particular, the changes in the amount of the light-green component determine the magnitude of increase in PA lumen in type B response.

Autopsy specimens of patients with CTEPH have revealed that thromboembolic material can be found in several elastic PAs at a wide range of diameters, starting from 0.5 mm [21,22]. Histological examinations of material harvested during PEA has uncovered the complex structure of organized thrombi, composed of fresh thrombotic material, and fibrotic tissue comprised of collagen, fibroblasts and inflammatory cells [23]. Some of the lesions resemble atherosclerotic plaques typical for systemic circulation with a core rich in cholesterol, increased cellularity, angiogenesis, and calcifications [24]. In our study, utilizing IVUS technology, we were able to visualize thromboembolic lesions in several elastic PAs, with diameters ranging from 2.2 to 8.4 mm. By implementing the VH technique, we found that the structure of the organized thrombi is heterogeneous, and includes four major components, namely, dark-green, light-green, red, and white components. 

In previous studies, IVUS-VH imaging has been validated ex-vivo and in-vivo as a reliable tool for the assessment of the composition and structure of atherosclerotic plaques occupying coronary arteries [25,26,27]. The technique uses an analysis of a backscatter radiofrequency signal during IVUS examinations to distinguish several components of the plaque. Several other studies in peripheral arterial vessels and saphenous vein grafts [28] confirmed the utility of IVUS-VH to correctly characterize plaque morphology [29]. To date no descriptions of IVUS-VH in CTEPH has been published.

In a group of patients, in whom PA lumen dilation depended on the compression of organized thrombi, we found that the light-green component was the most compressible part of the thrombotic lesions. This might therefore generate a hypothesis that lesions rich in this component are more suitable for BPA than lesions of other types, and thus can be treated with lower inflation pressures, perhaps making the procedure safer. This assumption, however, requires further validation, and would be of clinical importance since to date there has been no consensus on the specific technical approaches to be taken during BPA, especially in terms of the preferred compliance profile of the balloon catheters, and the optimal pressure of balloon inflation [30].

Our study adds to the current understanding of the mechanisms of BPA. Previously, Shimokawahara et al. [10], based on IVUS imaging, found that vessel stretching was the main cause of lumen enlargement, with only a minor contribution resulting from compression of the organized thrombi. We confirmed this observation but only in vessels of larger calibers. In contrast, in smaller arteries, it was the compression of the organized thrombi which was mainly responsible for lumen enlargement. Therefore, the differing findings from Shimokawahara et al. and our group might arise as a result of the caliber of the assessed vessels under study. The mean CSA of the treated PA segments in our study was 13.6 [9.7–20.2] mm^2^ as compared to the 23.2 ± 12.9 mm^2^ segments reported on by Shimokawahara et al. The differing mechanisms of lumen dilation after BPA in smaller and larger PA arteries may stem from longitudinal changes of the PA wall structure. The proximal segments of PA are composed mostly of elastic and collagen fibers, whose prevalence decreases gradually in favor of muscle fibers in more distal vessels [31]. Consequently, the elasticity of the PA wall decreases in parallel with the decrease in vessel diameter. We acknowledge that the type of response to BPA may be related to several individual characteristics of a patient. However, we found that almost all patients presented both types (A and B) of responses (60 [33–67]% of Type A response per patient) and there was only one patient who presented only Type A response in all assessed PA segments.

Experimental studies in vivo showed that elastic vessels respond to stretching with vessel dilation. The distending pressure is transferred to the elastic fibers, at which point it is directed from the elastic to the collagen fibers. In contrast, in muscular arteries (which are less elastic), the distending pressure is transferred directly to the organized thrombi leading to its compression [32,33].

### Strengths

Our study has several strengths. Firstly, we have distinguished differing mechanisms of lumen dilation after BPA, which depend on the diameter of the treated vessel. Secondly, we have elucidated, for the first time, the IVUS-VH structure of the organized thrombi occupying the PA segments. Lastly, we have found that the structure of the organized thrombi affects its compressibility, a finding which may well have important clinical meaning. 

## 5. Limitations

Our study has several limitations. First of all, only a moderate number of patients was enrolled. To overcome this, we assessed several arterial segments in each patient. Secondly, we analyzed only a subset of all the stenosed PA segments which had been subjected to BPA as a result of the application of quality control criteria for the acceptance of adequate images for further assessment. In particular, and similar to other authors, we excluded from the analysis any segments for which a steady position for the IVUS probe was not achievable [10].

## 6. Conclusions

The dominant mechanism of lumen dilation after BPA in CTEPH patients depends on the diameter of the treated segment: Wall stretching dominates in larger PAs and compression of the organized thrombi in smaller. Use of the IVUS-VH technique allowed us to identify the composition of the organized thrombi, which in turn, determined its susceptibility to compression during BPA. 

## Figures and Tables

**Table 1:** Chronic thromboembolic pulmonary hypertension (CTEPH) patients clinical characteristics (*n* = 20).

Variable				
Age [years]	67	65–75		
Male sex [*n*,%]	6	30		
Time from onset of symptoms to CTEPH diagnosis [months]	10	5–22		
	Before BPA		6-months after final BPA	
NYHA class [*n*, %]				
III	20	100	2	10
II	0	0	12	60
I	0	0	6	30
6-min walking distance [m]	330	260–380	393	34–450
NT-proBNP [pg/mL]	1726	521–2678	236	144–722
mPAP [mmHg]	39	37–50	29	25–31
RAP [mmHg]	4	3–7	4	3–6
CI [L/min/m^2^]	2.31	1.95–2.62	2.49	2.32–3.00
PVR [WU]	8.9	6.3–11.1	3.9	3.5–5.7

Continuous variables are presented as medians and interquartile ranges. Abbreviations: BPA—balloon pulmonary angioplasty, CI—cardiac index; CTEPH—chronic thromboembolic pulmonary hypertension; mPAP—mean pulmonary artery pressure; NT-proBNP—N-terminal prohormone of brain natriuretic peptide; PVR—pulmonary vascular resistance; RAP—right atrial pressure.

**Table 2:** Comparison of organized thrombi structure for type A and type B mechanisms of balloon pulmonary angioplasty (BPA).

		Type A *n* = 49	Type B *n* = 49	*p*
Lesion type [*n*, %]	ring	7 [14]	6 [12]	0.74
	web	42 [86]	43 [88]	
Lesion location [*n*, %]	segmental	21 [43]	15 [31]	0.34
	subsegmental	28 [57]	34 [69]	
Balloon to segment ratio [%]		80.5 [59.8–88.3]	82.7 [68.4–93.0]	0.4
CSA [mm^2^]:	L-CSA	5.25 [3.8–6.2]	3.85 [2.7–5.3]	0.03
	E-CSA	15.25 [11.7–23.3]	12.6 [8.1–18.4]	0.01
	T-CSA	11.2 [7.3–17.3]	8.3 [5.1–13.8]	0.01
Components of the organized thrombus in IVUS-VH [%]	Dark-green	55.75 [47.8–59.8]	59.6 [53.9–66.8]	0.02
	Light-green	35.15 [27.3–44.1]	27.4 [19.5–42]	0.03
	Red	6.2 [3.4–11.7]	7.85 [4.7–17]	0.11
	White	0.35 [0–1.7]	0.15 [0–0.7]	0.1
Change (∆) in CSA of the stenosed PA after BPA [mm^2^]:	∆ L-CSA	1.9 [1.2–3.4]	0.8 [0.6–1.7]	0.001
	∆ E-CSA	2.8 [1.7–4.6]	0.1 [−0.4–0.7]	0.001
	∆ T-CSA	0.7 [−0.1–2.0]	−0.9 [−2.0–−0.1]	0.001
Percentage change (∆) in CSA of the stenosed PA after BPA [%]:	∆ L-CSA	37.4 [21.4–64.1]	27.8 [15.0–39.4]	0.05
	∆ E-CSA	20.4 [10.1–30.4]	0.7 [−2.1–4.5]	0.001
	∆ T-CSA	7.0 [−1.3–28.9]	−13.4 [−28.3–−1.9]	0.001

A stretch response was assigned to lesions when any increase in the stenosed PA segment CSA was greater than the median value of the change in the stenosed PA segment CSA after BPA. Abbreviations: CSA—cross-sectional area; E-CSA—pulmonary artery stenosed segment cross-sectional area; L-CSA—lumen cross-sectional area; T-CSA—organized thrombus cross-sectional area.

**Table 3:** Multivariable logistic regression analysis for the prediction of stretch response (type A mechanism) to balloon pulmonary angioplasty (BPA).

Variable	OR	95% CI	*p*
Dark-green CSA (%)	0.98	0.91–1.06	0.68
Light-green CSA (%)	1.01	0.95–1.07	0.70
E-CSA (mm^2^)	1.09	1.01–1.17	0.03
Organized thrombus CSA (%)	0.98	0.94–1.02	0.40
Balloon to vessel diameter ratio (%)	1.01	0.99–1.03	0.19

Abbreviations: CI—confidence interval; CSA—cross-sectional area; E-CSA—pulmonary artery stenosed segment cross-sectional area; OR—odds ratio.

## Supplementary Materials

The following are available online at https://www.mdpi.com/2077-0383/9/6/1655/s1, Table S1. Structure of organized thrombi in patients treated with targeted therapies (treprostinil, sildenafil, riociguat) and in patients who did not use pulmonary hypertension specific pharmacotherapy, Table S2. Correlations between structure of organized thrombus and markers of hemodynamic severity of chronic thromboembolic pulmonary hypertension, Figure S1. Bland–Altman graphs showing intraobserver and interobserver (2 observers) agreement for the assessment of the amount of dark-green, light-green, red, and white components in 15 pulmonary artery segments. The solid line represents observed average agreement. The dashed lines represent 95% limit of agreement. Measurements are considered repeatable if 95% of the differences are within 2 SD [20].
