# Supplementary material for: Virtual Histology to Evaluate Mechanisms of Pulmonary Artery Lumen Enlargement in Response to Balloon Pulmonary Angioplasty in Chronic Thromboembolic Pulmonary Hypertension
Source: J Clin Med. 2020 Jun 1;9(6):1655. doi: 10.3390/jcm9061655 (PMC7355673; doi:10.3390/jcm9061655)
Supplement: Supplementary file 1 [file jcm-09-01655-s001.zip › SupplementalTables.docx]

| Table S1. Structure of organized thrombi in patients treated with targeted therapies (treprostinil, sildenafil, riociguat) and in patients who did not use pulmonary hypertension specific pharmacotherapy. | | | |
| --- | --- | --- | --- |
|  | Treprostinil and/or sildenafil and/or riociguat | No treatment | p |
| Dark-green (%) | 57.3 [46.6 - 65.2] | 56.7 [52.3 - 64.8] | 0.57 |
| Light-green (%) | 34.2 [20.5 - 49.5] | 32.8 [26.4 - 43.8] | 0.66 |
| Red (%) | 6.8 [2.4 - 11.9] | 5.9 [4.1 - 10.3] | 0.71 |
| White (%) | 0.1 [0 - 0.9] | 0.2 [0 - 0.8] | 0.47 |
|  | Treprostinil | No treatment |  |
| Dark-green (%) | 57.8 [47.7 - 65.8] | 56.45 [51.7 - 64.1] | 0.9 |
| Light-green (%) | 32.3 [20.5 - 48.1] | 34.1 [26.4 - 44.1] | 0.99 |
| Red (%) | 7 [2.4 - 11.9] | 5.55 [3.9 - 10.3] | 0.96 |
| White (%) | 0.1 [0 - 0.9] | 0.2 [0 - 0.8] | 0.59 |
|  | Riociguat or sildenafil | No treatment |  |
| Dark-green (%) | 57.95 [45.75 - 61.15] | 56.7 [48.3 - 65.1] | 0.99 |
| Light-green (%) | 34.8 [23.05 - 49.5] | 32.3 [21 - 44.9] | 0.65 |
| Red (%) | 5.6 [2.2 - 11.7] | 6.6 [3.4 - 11.4] | 0.45 |
| White (%) | 0.05 [0 - 0.9] | 0.2 [0 - 0.9] | 0.46 |

| Table S2. Correlations between structure of organized thrombus and markers of hemodynamic severity of chronic thromboembolic pulmonary hypertension. | | | | | | | | |
| --- | --- | --- | --- | --- | --- | --- | --- | --- |
|  | Dark-green (%) | | Light-green (%) | | Red (%) | | White (%) | |
|  | r | p | r | p | r | p | r | p |
| mPAP | -0.01 | 0.93 | 0.13 | 0.14 | -0.11 | 0.23 | -0.08 | 0.38 |
| PVR | 0.09 | 0.28 | 0.00 | 1.00 | -0.03 | 0.70 | 0.02 | 0.86 |
| CI | -0.06 | 0.53 | 0.02 | 0.79 | 0.04 | 0.66 | -0.05 | 0.60 |
| RAP | 0.04 | 0.67 | 0.03 | 0.75 | -0.07 | 0.46 | 0.07 | 0.40 |
| CI – cardiac index; mPAP – mean pulmonary artery pressure; PVR – pulmonary vascular resistance; RAP – right atrial pressure | | | | | | | | |
