# Supplementary figures and images for: Virtual Histology to Evaluate Mechanisms of Pulmonary Artery Lumen Enlargement in Response to Balloon Pulmonary Angioplasty in Chronic Thromboembolic Pulmonary Hypertension
Source: J Clin Med. 2020 Jun 1;9(6):1655. doi: 10.3390/jcm9061655 (PMC7355673; doi:10.3390/jcm9061655)

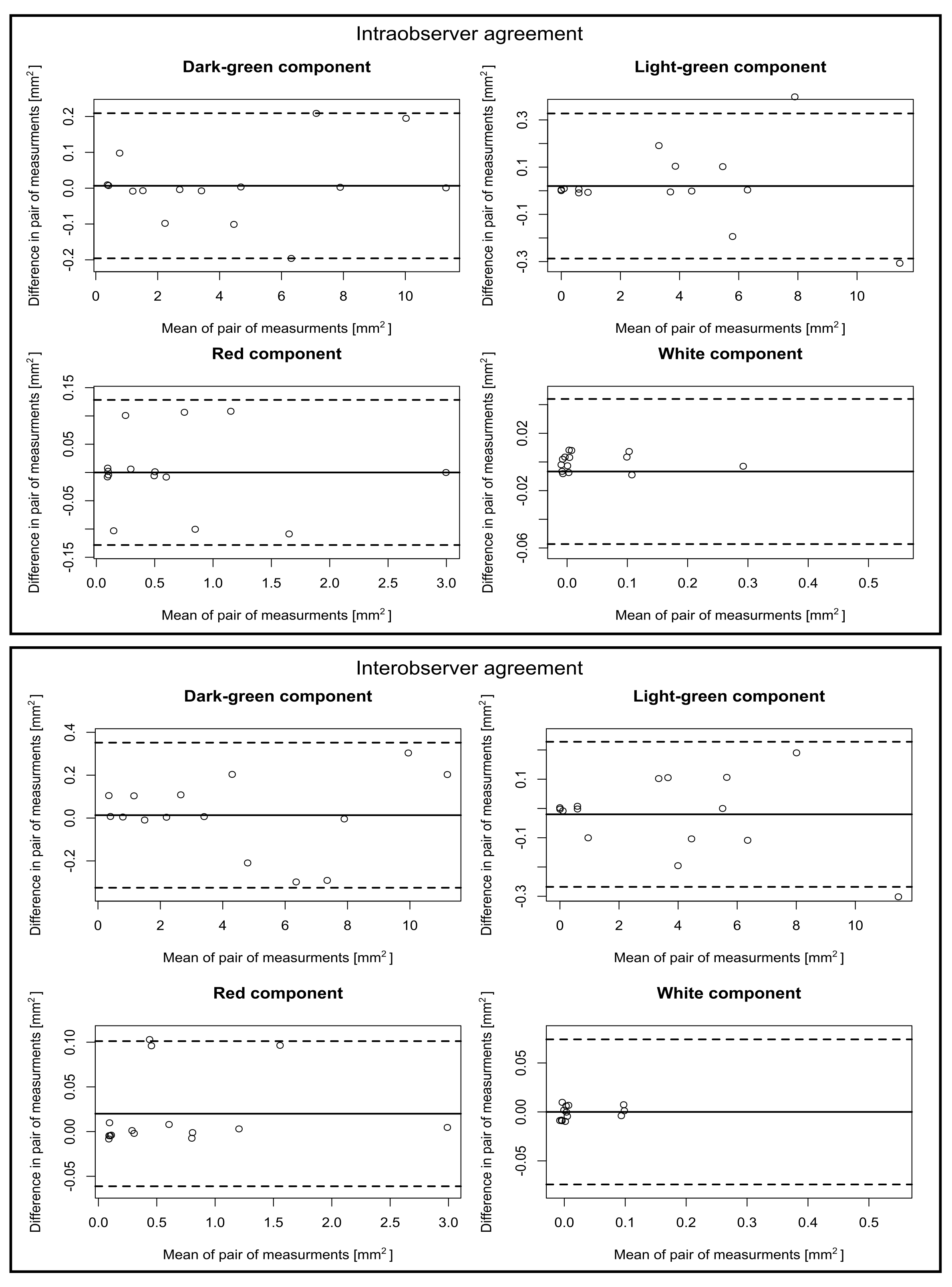

Supplement: Supplementary file 1 [file jcm-09-01655-s001.zip › SupplementalFigureS1.png]
